# An evolutionary divergent pestivirus lacking the N^pro^ gene systemically infects a whale species

**DOI:** 10.1080/22221751.2019.1664940

**Published:** 2019-09-17

**Authors:** Wendy K. Jo, Cornelis van Elk, Marco van de Bildt, Peter van Run, Monique Petry, Sonja T. Jesse, Klaus Jung, Martin Ludlow, Thijs Kuiken, Albert Osterhaus

**Affiliations:** a Research Center Emerging Infections and Zoonoses (RIZ), University of Veterinary Medicine Hannover, Hannover, Germany; b Department Viroscience, Erasmus MC Rotterdam, Rotterdam, The Netherlands; c Institute for Animal Breeding and Genetics, University of Veterinary Medicine Hannover, Hannover, Germany

**Keywords:** Pestivirus, porpoise, systemic, marine mammal, N^pro^

## Abstract

Pestiviruses typically infect members of the order Artiodactyla, including ruminants and pigs, although putative rat and bat pestiviruses have also been described. In the present study, we identified and characterized an evolutionary divergent pestivirus in the toothed whale species, harbour porpoise (*Phocoena phocoena*). We tentatively named the virus Phocoena pestivirus (PhoPeV). PhoPeV displays a typical pestivirus genome organization except for the unique absence of N^pro^, an N-terminal autoprotease that targets the innate host immune response. Evolutionary evidence indicates that PhoPeV emerged following an interspecies transmission event from an ancestral pestivirus that expressed N^pro^. We show that 9% (*n* = 10) of stranded porpoises from the Dutch North Sea coast (*n* = 112) were positive for PhoPeV and they displayed a systemic infection reminiscent of non-cytopathogenic persistent pestivirus infection. The identification of PhoPeV extends the host range of pestiviruses to cetaceans (dolphins, whales, porpoises), which are considered to have evolved from artiodactyls (even-toed ungulates). Elucidation of the pathophysiology of PhoPeV infection and N^pro^ unique absence will add to our understanding of molecular mechanisms governing pestivirus pathogenesis.

## Introduction

Pestiviruses are enveloped viruses that belong to the genus *Pestivirus* within the family *Flaviviridae*. They have a wide host range and are responsible for significant levels of morbidity and mortality worldwide in mammals of the order Artiodactyla, which include ruminants and pigs. The typical pestivirus species are classical swine fever virus (CSFV) in pigs, bovine viral diarrhoea virus 1 and 2 (BVDV-1 and BVDV-2) in cattle, and border disease virus (BDV) in sheep [1]. Atypical pestiviruses have also been found in wild ruminants, such as antelopes and giraffes [2,3]. In the past two decades, improved technology and better surveillance have facilitated the detection of new members of the genus *Pestivirus*, in some cases leading to expansion of known host species [4]. In pigs, three novel pestiviruses evolutionarily distant to CSFV have been identified: Bungowannah pestivirus, atypical porcine pestivirus (APPV), and most recently the lateral shaking inducing neurodegenerative agent (LINDA) virus. Recent metagenomics studies have indicated that pestiviruses are not restricted to Artiodactyla species, as putative bat pestiviruses (BatPeV) and rat pestiviruses have been discovered [5,6], along with a pestivirus-like virus in a soybean cyst nematode [7].

Pestiviruses have two biotypes, cytopathic (cp) and non-cytopathic (ncp) viruses. The latter generate persistent infections without overt damage and are the most common forms found in the field [8]. Infected animals display mostly inapparent or mild symptoms. However, animals with acute infection can develop severe disease, characterized by haemorrhages, respiratory failure, gastrointestinal problems, and central nervous system disorders [4]. Recombination events upstream viral protein NS3 appear to trigger the cleavage of NS2-NS3 by autoprotease activity exerted by NS2, after which the virus does become cytopathic [9].

The genome of pestiviruses consists of a single molecule of positive-sense single-stranded RNA of about 12.3 kb that encodes one long open reading frame of about 3900 amino acids (aa) [10]. Pestiviruses are distinguished from other flavivirus genera by the unique presence of N^pro^ and E^rns^, which are both essential in antagonizing mediators of the host innate immune response [8]. The autocatalytic N-terminal protease N^pro^ is present at the beginning of the polyprotein and targets the host interferon regulatory factors 3 and 7 [11,12]. The glycoprotein E^rns^ possesses RNase activity influencing the interferon response.

Investigation into strandings of harbour porpoises (*Phocoena phocoena*) along the Dutch North Sea coast over a 15 year period was undertaken to identify morbidity and mortality factors, including possible underlying viral aetiologies. Previous studies have reported a limited number of novel viruses in these marine mammals that are heterologous to viruses of terrestrial mammals, including porpoise morbillivirus [13], norovirus [14], herpesviruses [15] and adenovirus [16]. We report the identification and characterization of a novel divergent pestivirus and demonstrate that the presence of N^pro^ is not a prerequisite for a pestivirus to infect its host.

## Materials and methods

### Animals and post-mortem examination

The samples used in this study were obtained from wild harbour porpoises that had stranded dead or alive along the Dutch North Sea coast. The live ones had been taken to the Dutch rehabilitation centre SOS Dolfijn (application number FF/75/2012/036) where they survived for variable periods of time. Animals that died or had to be euthanized were autopsied at Erasmus Medical Center. Tissue samples were fixed in 10% neutral-buffered formalin, embedded in paraffin and used for diagnostic purposes to determine the cause of stranding. Additional samples from the same organs were also frozen at −80°C for virological investigations.

### Next generation sequencing

Lung and brain tissue samples of three harbour porpoises (NS170385-87), suspected with encephalitis upon clinical observation by a veterinarian and diagnosed post-mortem by histological examination, were processed for Next generation sequencing (NGS). Briefly, 25-105 mg of tissue were lysed in 500 μL of PBS using ceramic beads in a FastPrep-24 5G homogenizer (MP Biomedical), followed by 3× freeze/thaw cycles. Homogenates were centrifuged and passed through a 0.45 μm filter. RNA was isolated using TRIzol (Thermo Fischer Scientifics, Waltham, MA, USA) and transcribed to cDNA using a mix of random and non-ribosomal hexamers [17] by Superscript IV (Thermo Fischer Scientifics). Second strand cDNA was generated by Klenow fragment (New England Biolab [NEB], Ipswich, MA, USA). Random amplification of samples was performed following a sequence-independent, single-primer amplification protocol [18]. PCR products were purified and the DNA library was prepared according to NEBNext^®^ Ultra™ II DNA Library Prep Kit protocol (NEB) and subsequently sequenced on an Illumina MiSeq system with the MiSeq Reagent Kit v3 (2 × 300 bp paired-end; Illumina).

### Generation of full-length genome sequence

NGS raw data were analysed using a previously developed metagenomics pipeline as described [19]. Quality-trimmed reads were mapped to DNA and peptide viral sequences database retrieved from GenBank using Bowtie v2.2.9 [20] and Pauda v1.0.1 [21]. Quality trimming of raw data and *de novo* assembly of contigs larger than 500 bp was followed using the software CLC Genomics Workbench v11 (CLC Bio, Aarhus, Denmark). Contigs were also mapped against non-redundant protein sequences database (blastx) using the same software. All contigs with similarity hits to pestiviruses were retrieved and an assembly was performed using SeqMan Pro (LaserGene software package, DNAStar, Madison, WI). To confirm genome sequence generated by NGS data, primers were designed to amplify the complete genome of NS170385-Lung (Table S1, Supplementary Information). A rapid amplification of cDNA ends (RACE) protocol to determine 5′ genome end was used. Briefly, RNA was polyadenylated with a poly(A) polymerase (NEB) and transcribed to cDNA using a poly(T) adaptor flanking the 5′ end. A PCR was then performed with primers designed to target the newly inserted poly(T) tail as well as the 5′ region of the novel pestivirus genome generated by NGS data (Table S1, Supplementary Information).

### Phylogenetic analyses

Complete genome sequences of 53 pestiviruses representative of all identified pestivirus species found to date (*A*-*K*), were retrieved from GenBank database (Table S2, Supplementary Information). Alignment of nucleotide and protein sequences was conducted by MAFFTv7 [22]. Phylogenetic trees of the complete polyprotein and partial PhoPeV nucleotide genomes (5′UTR, C, Erns, and E2) were calculated using the maximum likelihood method in MEGA7.0 [23] with 1000 bootstraps. According to the Bayesian information criterion, LG + G + F was selected as best-fit model for the complete polyprotein, whereas TN93 + G was selected as best-fit model for the partial nucleotide genomes.

### Histopathological analysis and in situ hybridization

After fixation in 10% neutral-buffered formalin and embedding in paraffin, tissue sections from animals NS170385 and NS170386 were stained with haematoxylin and eosin for histopathological evaluation or with *in situ* hybridization (ISH) as described previously [15]. A probe targeting specific PhoPeV NS2-NS3 region was designed by Advanced Cell Diagnostics (Hayward, California, USA). ISH was performed using RNAscope 2.0/2.5 assay kit (Advanced Cell Diagnostics, Inc.) following manufacturer instructions for FFPE samples. In brief, 5-μm-thick tissue sections were deparaffinised in xylene and dehydrated in 100% ethanol. Slides were next pretreated to allow access to target RNA. The probe was subsequently added to slides and hybridized for 2 h at 40°C with six subsequent amplification steps. Signal was visualized with Fast Red. The section was counterstained with haematoxylin and mounted with Ecomount.

### Screening of PhoPeV in harbour porpoises

A PhoPeV-specific real-time reverse transcription PCR (qRT-PCR) was developed to screen for the novel pestivirus in stranded harbour porpoises from the North Sea. The primers and probe were designed to target the NS3 region of PhoPeV, with 5′-aaccatctgagtgtgaccttgagtc-3′ as forward primer, 5′-tcaatcaaccttcttggtagctcagtg-3′ as reverse primer, and 5′-tttaaacaagtgaccctggccaccgg-3′ as probe labelled with FAM-BHQ-1. Samples were homogenized, centrifuged and supernatants taken for RNA extraction. Automated sample processing was performed with a QIAcube instrument using the QIAmp Viral RNA Mini kit (Qiagen). A 45 cycle one-step qRT-PCR with annealing temperature of 57°C was carried out following the Luna Probe One-Step RT-qPCR kit (NEB) protocol. All available tissue samples from PhoPeV NGS-positive harbour porpoises were analysed using the newly developed qRT-PCR. An additional 109 kidneys from wild harbour porpoises that had stranded dead or alive along the Dutch North Sea coast and when alive had been nursed in the Dutch rehabilitation centre SOS Dolfijn for variable periods of time before dying, were also screened using this methodology. Spleen and brain tissue samples (if available) were also included from animals in which the kidney was found to be PhoPeV PCR-positive.

### Cell culture and virus isolation

PK-15 cells were cultured in DMEM media supplemented with 10% FBS and 1% penicillin/streptomycin. MDBK cells were cultured in advanced MEM media supplemented with 10% FBS, 1% penicillin/streptomycin and 1% GlutaMax. Before virus isolation attempts, cells were washed with warm media without FBS and diluted kidney homogenates of samples NS170385 and NS170386 were added to 90% confluent cells and incubated at 37°C with 5% CO_2_ for 1–1.5 h. Cells were then washed twice and incubated overnight in growth media with 1% FBS. Media was changed the next day. Cells were blind passaged after 3–4 days. Supernatant and cells were taken for PhoPeV-specific qRT-PCR analyses after each new passage.

## Results

### Identification of a novel pestivirus

**Figure 1.**
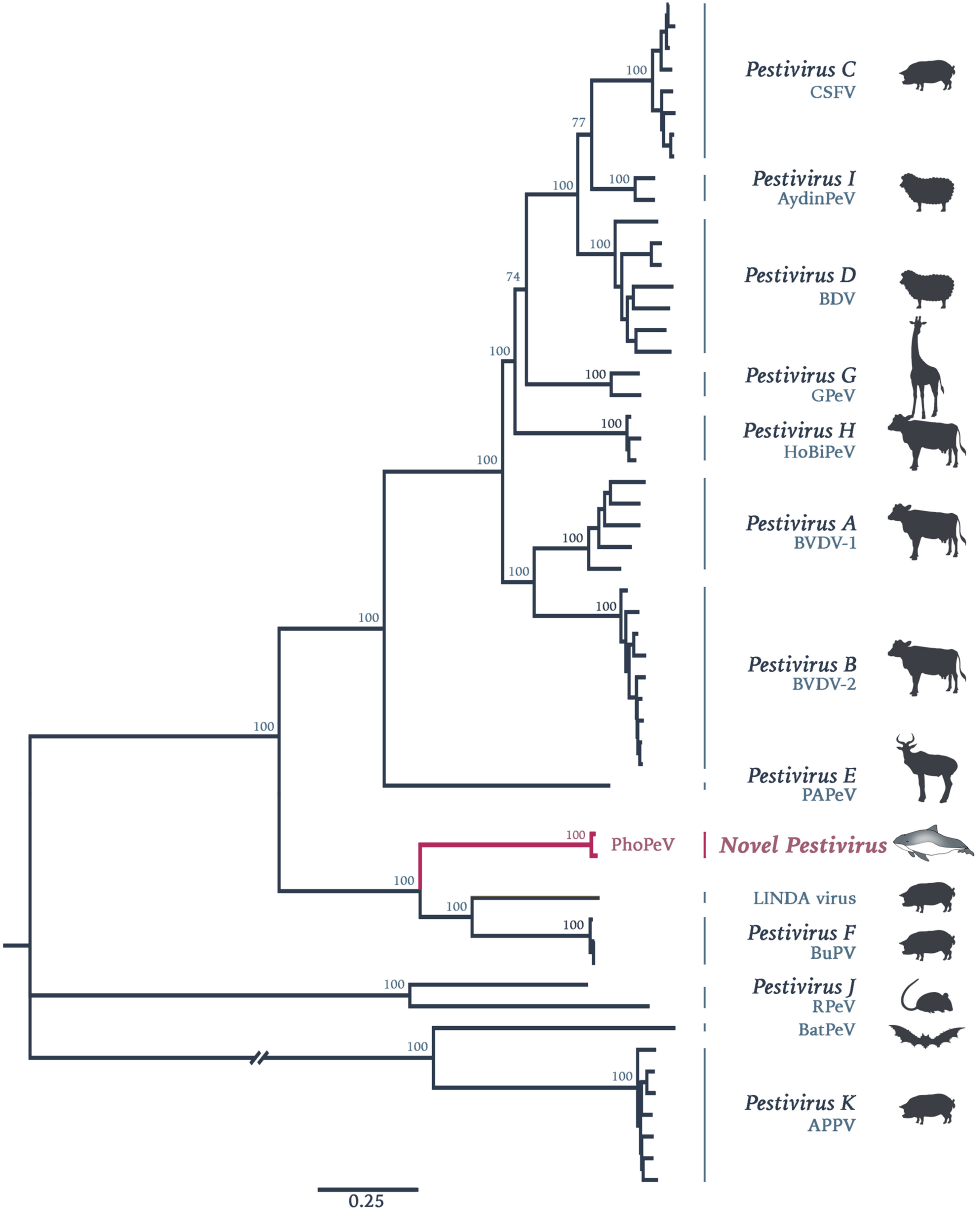
Phylogenetic reconstruction using maximum likelihood estimation of complete polyprotein of known and putative pestiviruses. Main bootstrap values are presented at nodes. Scale bar indicates number of amino acids changes per site. Taxon names are presented by the virus species and virus abbreviation. Abbreviations: CSFV, Classical swine fever virus; AydinPeV, Aydin-like pestivirus; BDV, Border disease virus; GPeV, Giraffe pestivirus; HoBiPeV, HoBi-like pestivirus; BVDV, Bovine viral diarrhea virus; PAPeV, Proghorn antelope pestivirus; PhoPeV, Phocoena pestivirus; LINDA, Lateral shaking inducing neurodegenerative agent; BuPV, Bungowannah porcine pestivirus; RPeV, Rat pestivirus; BatPeV, Bat pestivirus; APPV, Atypical porcine pestivirus

Lung and brain samples from three harbour porpoises with encephalitis indicative of viral infection were selected for NGS. Data was first analysed using a metagenomics pipeline [19], the results of which indicated the presence of a virus with homology to BVDV at the protein level in two of the animals (Figure S1, Supplementary Information). Assembly of contigs from these reads resulted in the discovery of a 11,880 bp sequence of a novel pestivirus, tentatively named Phocoena pestivirus (*PhoPeV*)*.* The 5′ end of the new virus was determined by RACE due to low coverage in this region. The complete PhoPeV genome sequence (GenBank accession nos. MK910227-29) was corroborated with Sanger sequencing data based on primers designed from the NGS reads. The two newly generated full-length genomes from the harbour porpoises differ 97.6% in their genome. Sequence alignment of known pestivirus species indicated that PhoPeV is most related to the porcine pestiviruses Bungowannah virus and LINDA virus with approximately 60% homology at the amino acid level, and only about 27% homology to the more divergent BatPeV and APPV. Phylogenetic analysis using maximum likelihood estimations showed that PhoPeV also clusters with Bungowannah and LINDA viruses, forming a monophyletic group distantly related to other typical pestiviruses (Figure 1).

### Absence of putative N^pro^ coding region from the PhoPeV genome

**Figure 2.**
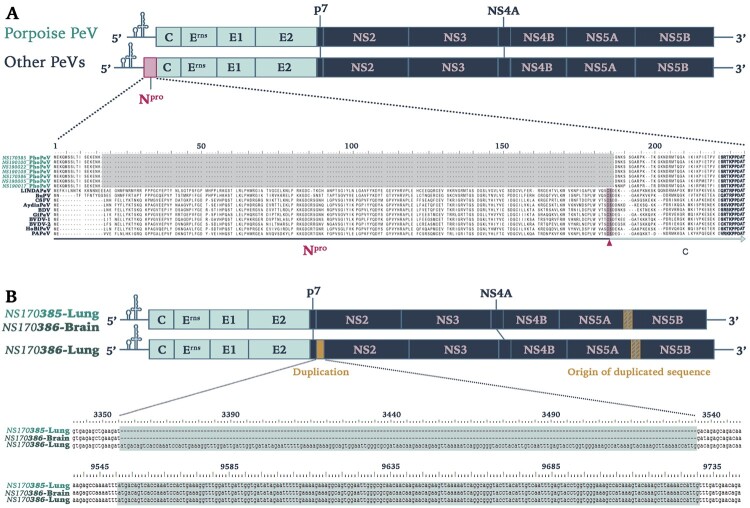
Genomic characterization of Phocoena pestivirus. (a) Comparison of PhoPeV to other pestivirus genome organization. PhoPeV genome arrangement is similar to other pestiviruses except for the unique absence of N^pro^ in screened harbour porpoises (*n* = 7). Alignment of N^pro^ area of PhoPeV and other pestivirus sequences is zoomed in. Absence of N^pro^ gene coding sequences is highlighted in grey, conserved area in the capsid (from 221nt) is in bold. C/S cleavage site between N^pro^ and Capsid is highlighted in pink and indicated with an arrowhead. Virus (Abbreviation – GenBank Accession No.): LINDA pestivirus (LINDAPeV-KY436034), Bungowannah pestivirus (BuPV-EF100713), Classical swine fever (CSFV-X87939), Aydin-like pestivirus (AydinPeV-JX428945), Border disease virus (BDV-AF037405), Giraffe pestivirus (GiPeV-AD144617), Bovine viral diarrhea virus-1 (BVDV-1-M31182), Bovine viral diarrhea virus-2 (BVDV-2-U18059), HoBi-like pestivirus (HoBiPeV-AB871953), Proghorn pestivirus (PAPeV-AY781152). (b) Comparison between PhoPeV sequences in different organs generated from two animals. The lung of animal NS170386 has an insertion (yellow) between p7 and NS2, which sequences originates from a region between NS5A and NS5B (striped yellow square). Duplicated and origin of duplicated sequence are zoomed in and are highlighted in teal colour.

PhoPeV has a polyprotein size of 3762 aa, which makes it smaller than other pestiviruses by approximately 150 aa, but similar in size with BatPeV (3663 aa) and APPV (3635 aa). The untranslated regions (UTRs) were of similar size to those of other pestiviruses, with a 5′UTR of 382 bp and 3′UTR of 212 bp. The most striking feature of the PhoPeV genome was the absence of N^pro^ sequences (Figure 2a), as the alignment with other pestivirus species showed a gap at the start of the polyprotein which normally encodes N^pro^, followed by presence of sequences homologous to C. All other putative structural and non-structural pestivirus proteins were identified. Cleavage sites were recognized based upon homology with Bungowannah virus. The cleavage sites used by NS3 were all conserved, having Leu at P1 and Ser/Ala at P1′ positions. Interestingly, it was also noted that PhoPeV sequences from the lung of animal NS170385 had a 180nt insertion at the start of NS2. The inserted sequence was homologous to a sequence encompassing the C-terminal region of NS5A and N-terminal region of NS5B (Figure 2b). The two variant sequences (with and without the insertion) were detected in the brain of the same animal (NS170386), although the variant without insertion was predominant according to the Sanger sequencing results (Figure S2, Supplementary Information).

### Phopev tropism characterized by qRT-PCR and ISH

**Figure 3.**
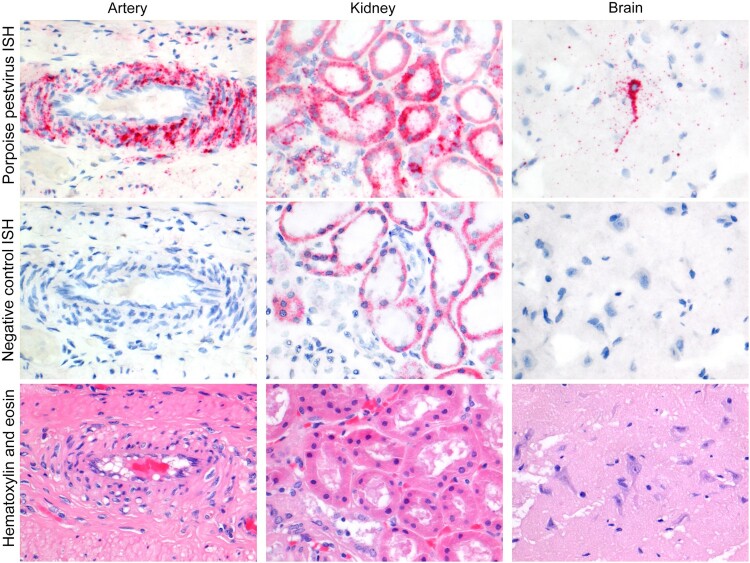
Phocoena pestivirus infects different cell types of harbour porpoises without histopathological changes. PhoPeV RNA expression is visible as bright red cytoplasmic staining in smooth muscle cells in the wall of an intestinal artery, epithelial cells in cortical tubules of the kidney, and neurons in the cerebrum of the brain, based on *in situ* hybridization (ISH) specific for Phocoena pestivirus (top row). Negative control ISH sections of these stain negative (middle row). Serial sections of these tissues, stained by hematoxylin and eosin, do not show any histopathological changes (bottow row). The narrow clefts in the neuropil of the brain are due to freeze-thaw artifact. Original objective magnifications for all panels: 40×. Artery and kidney were from porpoise NS170386, brain was from porpoise NS170385.

The two harbour porpoises positive by NGS for PhoPeV had a systemic infection, as all analysed tissues were positive by both ISH (Table 1) and qRT-PCR (Table 2). All tissues of both porpoises expressed pestivirus RNA (Table 2). Pestivirus RNA was visible by ISH as red staining in the cytoplasm, and ranged from single granules to multiple granules to diffuse staining of the cytoplasm (Figure 3). Pestivirus RNA expression was seen neither in negative control samples nor in tissues of a porpoise that was negative for pestivirus by PCR. The cell types that expressed pestivirus RNA were mainly smooth muscle cells and epithelial cells, but a variety of other cell types were also involved (Table 1). Positive smooth muscle cells were found in the walls of small and medium-sized arteries of every tissue examined, as well as in the aorta wall. In addition, positive smooth muscle cells were found in the muscular layers of digestive tract tissues, urinary bladder, bronchi and bronchioles, and in the trabeculae of the spleen. Positive epithelial cells were found in the mucosal lining of the respiratory, digestive, and urogenital systems. Specialized epithelial cell types expressing pestivirus RNA were keratinocytes, pancreatic acinar cells, bile duct epithelial cells, renal tubular epithelial cells, Sertoli cells, and thyroid follicle epithelial cells. Other cell types besides smooth muscle cells and epithelial cells also expressed PhoPeV RNA. These were neurons in the brain, cardiomyocytes in the heart, endocrine cells in the islets of Langerhans and adrenal gland, and mononuclear cells (tentatively identified as dendritic cells, lymphocytes, or both) in lymph nodes and spleen. Occasional mononuclear cells in hepatic sinusoids expressed pestivirus RNA and were tentatively identified as Kupffer cells.

**Table 1.** Cell types throughout the organ systems of two harbour porpoises (NS170385-86) infected by PhoPeV. Tissue sections were stained for PhoPeV RNA by *in situ* hybridization.

Organ system	Tissue	Cell types expressing pestivirus RNA
Nervous	Cerebrum	Neuron
	Cerebellum	Neuron
Cardiovascular	Heart ventricle	Cardiomyocyte
	Aorta^a^	Smooth muscle cell
	Artery	Smooth muscle cell
Respiratory	Blowhole^a^	Keratinocyte
	Trachea	Respiratory epithelial cell, submucosal gland epithelial cell
	Bronchus	Respiratory epithelial cell, submucosal gland epithelial cell, smooth muscle cell
	Lung	Bronchiolar smooth muscle cell, alveolar wall interstitial cell
Digestive	Lip^b^	Keratinocyte
	Esophagus	Surface epithelial cell
	Pharynx^a^	Surface epithelial cell
	Stomach	Gastric pit epithelial cell, smooth muscle cell
	Intestine	Enterocyte, smooth muscle cell
	Pancreas	Exocrine acinar cell, islet of Langerhans cell
	Liver	Bile duct epithelial cell, Kupffer cell
Urogenital	Kidney	Glomerular cell, tubular epithelial cell, collecting duct epithelial cell, pelvic epithelial cell
	Urinary bladder	Transitional epithelial cell, smooth muscle cell
	Uterus^a^	Surface epithelial cell
	Testis^b^	Sertoli cell, epididymal duct epithelial cell
Lymphoid	Spleen	Mononuclear cell, smooth muscle cell
	Lung-associated lymph node	Mononuclear cell
	Mesenteric lymph node	Mononuclear cell
Endocrine	Adrenal gland	Cortical cell, medullary cell
	Thyroid gland	Follicle epithelial cell
Integumentary	Skin	Keratinocyte
Musculoskeletal	Skeletal muscle^b^	Negative

^a^Porpoise NS170386 only.

^b^Porpoise NS170385 only.

**Table 2.** Lesions and levels of PhoPeV RNA (inversely correlated with Ct values) in the tissues of PhoPeV-positive harbour porpoises.

Host_ID	Date of stranding	Days in rehabilitation	Lesions (gross pathology/histology)	Sample material	RT-PCR (Ct)
NS170385*	May 2012	9	COD: bronchopneumonia associated with nematode infection, cerebrum polioencephalitis multifocal mild, hepatitis necrotizing multifocal acute marked. Incidental lesions: adrenalitis with eosinphilic intranuclear inclusions lip ulcus, blowhole ulcer, pyloric stomach focal gastritis	Lung	21.1
				Brain	26.5
				Liver	27.7
				Kidney	15.9
				Spleen	20.2
				Bladder	19.2
				Muscle	19.3
NS170386*	Dec 2008	12	COD: bronchopneumonia associated with parasitic and bacterial infection, marked emaciation. Incidental lesions: epiglottal ulcer, necrotizing pharyngitis, necrotizing ulcerative esophagitis, cholangitis ulcerative dermatitis.	Lung	18.2
				Brain	22.6
				Liver	19.6
				Kidney	16.4
				Spleen	18.8
				Bladder	17.6
				Muscle	21.1
				Skin	25.7
NS190005	Mar 2003	26	COD: bilateral keratoconjunctivitis, pneumonia associated wiht bacterial infection. Incidental lesions: pneumonia associated dermatitis	Kidney	32.5
				Spleen	37.3
				Brain	38.2
NS190017	Apr 2004	35	COD: bronchopneumonia associated wiht lungworm larvae, pneumonia associated with Aspergillus infection. Incidental lesions: ulcura on gential slit, rostral tip of palatum durum and on cornea of left eye, colonic crypt abcesses	Kidney	22.4
				Spleen	25.8
				Brain	24.1
NS190022	Jun 2001	10	COD: pneumonia associated with a bacterial infection. Incidental lesions: oesophageal ulcerations, parasitic infections of stomach and pulmonary artery pneumonia associated lesion lymphadenopathy, pleuritis and pericarditis	Kidney	17.3
				Spleen	19.0
NS190025	Jul 2001	0^#^	cerebellar haemorhage focal, oesophageal ulcertion multifocal, conjunctivitis, catarrhal mild, dermatitis	Kidney	37.0
				Spleen	Neg
				Brain	38.0
NS190026	Apr 2001	0^#^	advanced state of autolysis, no abnormalities detected	Kidney	34.5
				Spleen	36.7
NS190075	Jan 1998	13 years	COD: pneumonia, hyperplasia of the papilla vater causing obstruction of the pancreatic duct	Kidney	34.0
		8 months^+^		Spleen	Neg
				Brain	34.8
NS190100	Sep 2014	0^#^	pneumonia and dermatitis	Kidney	20.8
				Spleen	30.7
				Brain	30.8
NS190109	Jul 2005	8	COD: encephalitis associated with herpesvirus infection, bronchopneumonia associated with nematode infection. Incidental lesions: pulmonary arteritis associated with nematode infection	Kidney	17.1
				Spleen	23.0

Notes: COD: cause of death; Ct: cycle threshold.

*Samples were also analyzed by NGS.

^#^Dead stranded.

^+^Kept in zoo collection.

Comparison of sequential tissue sections stained either by haematoxylin and eosin (for histopathological analysis) or by ISH (for PhoPeV pestivirus RNA expression) did not show any evidence of histological lesions caused by PhoPeV infection (Figure 3). Specifically, cells that expressed pestivirus RNA did not show evidence of cellular damage, and positive cells did not co-localize with histological evidence of inflammation, haemorrhage, or necrosis.

### Phopev isolation in cell culture

Isolation of PhoPeV was performed with kidney homogenates of the two NGS-positive animals with the cell lines MDBK (bovine) and PK-15 (porcine). Virus replication was confirmed by qRT-PCR and NGS of PhoPeV isolate (NS170385k). Virus isolations were assessed by detection of PhoPeV viral load using qRT-PCR as the virus did not appear to generate cytopathic changes. After three passages in MDBK cells, Ct values of supernatant from cells infected with PhoPeV/NS170386k were reduced from 33 to 22, indicating virus replication. In contrast, the amount of virus present in the supernatant of MDBK cells infected with PhoPeV/NS170385k homogenate remained in the same range throughout the three passages (Ct of 26). Similarly, after two passages in PK-15 cells, Ct values in supernatant dropped from 25 to 21 for PhoPeV/NS170386k-infected cells, whereas Ct values in supernatant were maintained at approximately 22 for PhoPeV/NS170385k-infected cells. Sequencing of supernatant and infected PK-15 cells with PhoPeV/NS170385 P2 by NGS confirmed the presence of PhoPeV.

### Prevalence and tissue distribution of PhoPeV among stranded North Sea harbour porpoises

**Figure 4.**
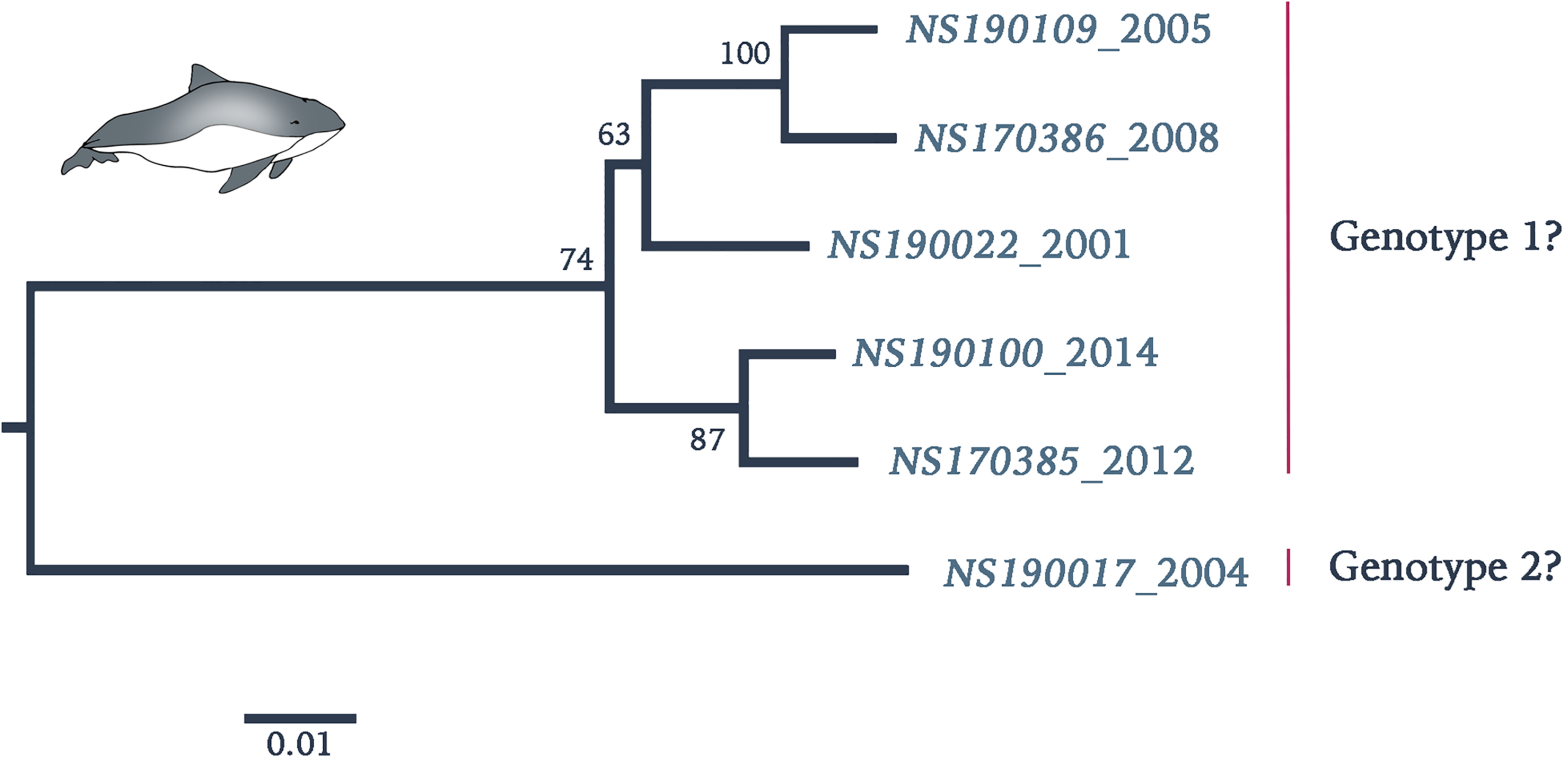
Phylogenetic reconstruction using maximum likelihood estimation of partial PhoPeV nucleotide genomes. Two main clades are highlighted as probable genotype 1 and 2. The PhoPeV nucleotide genomes used for analysis were 5′UTR, C, Erns, and E2 (GenBank accession nos. MK910230-37). Bootstrap values are presented at nodes. Scale bar indicates number of nucleotide changes per site LINDA virus was used as outgroup (GenBank accession number KY436034).

Retrospective screening by qRT-PCR of stranded harbour porpoises along the Dutch North Sea coast in the period 2001–2014 showed a total of 10 out of 112 (9%) animals positive for PhoPeV infection (Table 2). All other animals, which had died in the rehabilitation centre, including the ones that had been in direct contact with the positive animals, tested negative for pestivirus RNA in their tissues. Sequences between 5′UTR and C confirmed absence of N^pro^ sequences in 7 out of 10 samples (Figure 2a). Three of these samples (NS190025-27) had relatively high Ct values between 34 and 37, therefore absence/presence of N^pro^ sequences could not be corroborated. Analysis of concatenated 5′UTR, C, Erns, and E2 protein sequences (GenBank accession nos. MK910230-37) of a number of PhoPeV PCR-positive animals indicated that different strains and two probable genotypes were present among harbour porpoises from the North Sea in this time period (Figure 4). Pairwise sequence identity between strain NS170017 and other PhoPeV strains showed 10% difference at the nucleotide level.

## Discussion

Pestiviruses remain a major economic burden to livestock industry, due to incomplete control of classical pestiviruses such as CSFV, BVDV and BDV in many regions of the world. In the present study, we report the identification and characterization of PhoPeV, a novel evolutionary divergent pestivirus that lacks N^pro^ and may cause a systemic infection in the harbour porpoise. In addition we show that 9% (10/112) of stranded harbour porpoises from the Dutch North Sea coast carried PhoPeV.

Although most pestiviruses have been found in artiodactyl species, and cetacean species such as porpoises are considered to have evolved from them, the phylogenetic relatedness to other pestiviruses would not support the emergence of PhoPeV through co-evolution with the respective host species, but rather an interspecies transmission in the not too distant past. Moreover, the evolutionary relationship of PhoPeV, Bungowannah and LINDA viruses remains compelling, as these other viruses have only been found in pigs from Australia and Austria, respectively. It remains to be determined whether there was virus transmission between pigs and harbour porpoises at some time in the past, or whether an intermediary host species has played a role in mediating transmission of the common ancestor of this pestivirus clade.

The identification of PhoPeV in two out of three porpoises with encephalitis appears to be coincidental rather than evidence of a causative role, as the virus was present in multiple cells of nearly all organs investigated with no preferential co-localization of viral RNA with brain lesions. Moreover, PCR tests for morbillivirus and herpesvirus, which have been previously associated with encephalitis [13,15], were negative (data not shown). Therefore, the aetiology of the co-incidental encephalitis remained elusive. Consequently, tissue tropism and lack of PhoPeV associated lesions in the two positive porpoises investigated, most closely corresponds with that of ncp pestivirus infections, with BVDV infection in persistently infected cattle as the most striking parallel. It was previously reported that all 38 tissues from lymphoid, digestive, respiratory, endocrine, urogenital, nervous, cardiovascular, hematopoietic, and integumentary systems of two calves with persistent BVDV infection expressed virus antigen by immunohistochemistry [24]. Positive cells included lymphocytes, dendritic-like cells, macrophages, epithelial cells, and muscle cells. However, in common with our study, presence of viral RNA in tissues was not associated with tissue lesions [24]. We did not observe virus-associated encephalitis or glomerulonephritis, as have been diagnosed in some clinically healthy cattle persistently infected with BVDV [25]. In cattle, intrauterine infection of the foetus with ncp BVDV may induce selective immunotolerance, resulting in persistent viral infection in the absence of an adaptive immune response. Such animals can shed virus throughout their lifetime and are considered an important source of infection in the population [26,27]. It is interesting to note that like in ruminants, the placenta barrier of cetacean species is a complete epitheliochorial one [28], that may create similar conditions favouring intrauterine infection of the foetus. We speculate that a similar mechanism could explain the widespread PhoPeV infection and lack of associated pathological changes in the two investigated porpoises. Animals with analogous PhoPeV infections may represent an important source of virus transmission to naïve porpoises in the wider population. Nevertheless, given that these results are based on two animals and only one PhoPeV genotype, it remains to be determined whether this represented a true persistent infection or rather a transient infection, and whether PhoPeV infection of naïve porpoises, like in BVDV in cattle, is associated with enhanced pathology. Answers to these questions would have major consequences for health status of the population at large.

The most intriguing aspect of PhoPeV is the absence of the N^pro^ gene. This protein acts as an antagonist of IRF3 and IRF7 in other pestiviruses [11,12] and is not considered essential for viral replication in cell culture [29,30], as its replacement with a murine ubiquitin gene in a recombinant CSFV strain did not affect viral replication in SK-6 cells. However, when this recombinant virus was used to infect pigs, no disease symptoms were displayed while high antibody titres were later detected [31]. In addition, it has been shown that complete deletion of N^pro^ greatly compromises the growth rate of recombinant BVDV [32]. However, sequences directly downstream of the translation initiation codon have been suggested to be essential to pestivirus viability [33]. Therefore, in a recent study, the first four aa of N^pro^ were retained upstream of the capsid gene, instead of a complete deletion of the N^pro^ gene. This resulted in a slight reduction in the viral growth rate without compromising viability of the virus [34]. In the same study, recombinant strains of BVDV with either single or the combined mutations (almost complete deletion of N^pro^ and/or deletion of codon 349 that abrogates E^rns^ RNase activity) were used in pregnant cattle to evaluate their role in pestivirus persistent infection. Results indicated that only pregnant cattle infected with the double mutant strain cleared the infection, whereas virus reached foetuses and caused infection when inoculated with either wildtype or single mutant strains [34]. The ability of intact E^rns^ or another PhoPeV protein to antagonize the porpoise innate immune system and thus mediate systemic virus spread remains to be determined. Alternatively, unique features of the cetacean immune system may have supported a loss of N^pro^ from the ancestral PhoPeV. A loss of the *Mx1* and *Mx2* genes has been reported in toothed whales [35], the suborder to which the harbour porpoise belongs. *Mx* genes are important antiviral proteins, expression of which is regulated by type I interferon system, which in turn is controlled by IRF3 and IRF7 [36]. It remains to be elucidated whether the loss of N^pro^ from PhoPeV is associated with differences in the innate immune response of cetaceans, such as the absence of *Mx* genes or any other interferon-stimulated gene.

The identification of PhoPeV as a novel putative member of the genus *Pestivirus* in a cetacean species expands the host range potential of pestiviruses, and clearly warrants further studies into the susceptibility of other cetacean species to PhoPeV or other related pestiviruses. The observed natural deletion of N^pro^ from the PhoPeV genome highlights the genetic plasticity of pestiviruses and suggests that at least in one marine mammal species, systemic spread of a pestivirus is not predicated on the expression of N^pro^. Further research into the cetacean immune system and the role of N^pro^ during pestivirus infections will help dissect mechanisms underlying the evolution and pathogenesis of this unique virus.

## Supplementary Material

Supplemental Material

## Data Availability

The sequences generated in this study from two full-length PhoPeV genomes have been deposited under GenBank accession numbers (MK910227-37). Other data are available upon request.

## References

[1] Becher P, Orlich M, Shannon AD, et al. Phylogenetic analysis of pestiviruses from domestic and wild ruminants. J Gen Virol. 1997;78:1357–1366. doi: 10.1099/0022-1317-78-6-1357 9191930

[2] Becher P, Orlich M, Kosmidou A, et al. Genetic diversity of pestiviruses: identification of novel groups and implications for classification. Virology. 1999;262:64–71. doi: 10.1006/viro.1999.9872 10489341

[3] Vilcek S, Ridpath JF, Van Campen H, et al. Characterization of a novel pestivirus originating from a pronghorn antelope. Virus Res. 2005;108:187–193. doi: 10.1016/j.virusres.2004.09.010 15681069

[4] Blome S, Beer M, Wernike K. New leaves in the growing tree of pestiviruses. Adv Virus Res. 2017;99:139–160. doi: 10.1016/bs.aivir.2017.07.003 29029724

[5] Wu Z, Ren X, Yang L, et al. Virome analysis for identification of novel Mammalian viruses in bat species from Chinese Provinces. J Virol. 2012;86:10999–11012. doi: 10.1128/JVI.01394-12 22855479 PMC3457178

[6] Firth C, Bhat M, Firth MA, et al. Detection of zoonotic pathogens and characterization of novel viruses carried by commensal Rattus norvegicus in New York City. MBio. 2014;5:e01933–14. doi: 10.1128/mBio.01933-14 25316698 PMC4205793

[7] Bekal S, Domier LL, Gonfa B, et al. A novel flavivirus in the soybean cyst nematode. J Gen Virol. 2014;95:1272–1280. doi: 10.1099/vir.0.060889-0 24643877

[8] Peterhans E, Schweizer M. Pestiviruses: how to outmaneuver your hosts. Vet Microbiol. 2010;142:18–25. doi: 10.1016/j.vetmic.2009.09.038 19846261

[9] Becher P, Tautz N. RNA recombination in pestiviruses: cellular RNA sequences in viral genomes highlight the role of host factors for viral persistence and lethal disease. RNA Biol. 2011;8:216–224. doi: 10.4161/rna.8.2.14514 21358277

[10] Tautz N, Tews BA, Meyers G. The molecular biology of pestiviruses. Adv Vir Res. 2015;93:47–160. doi: 10.1016/bs.aivir.2015.03.002 26111586

[11] Gottipati K, Holthauzen LMF, Ruggli N, et al. Pestivirus N^pro^ directly interacts with interferon regulatory factor 3 monomer and dimer. Diamond MS, editor. J Virol. 2016;90:7740–7747. doi: 10.1128/JVI.00318-16 27334592 PMC4988160

[12] Fiebach AR, Guzylack-Piriou L, Python S, et al. Classical swine fever virus N^pro^ limits type I interferon induction in plasmacytoid dendritic cells by interacting with interferon regulatory factor 7. J Virol. 2011;85:8002–8011. doi: 10.1128/JVI.00330-11 21680532 PMC3147952

[13] Kennedy S, Smyth JA, Cush PF, et al. Viral distemper now found in porpoises. Nature. 1988;336:21–21. doi: 10.1038/336021a0 3185717

[14] de Graaf M, Bodewes R, van Elk CE, et al. Norovirus infection in harbor porpoises. Emerg Infect Dis. 2017;23:87–91. doi: 10.3201/eid2301.161081 27983498 PMC5176230

[15] van Elk C, van de Bildt M, van Run P, et al. Central nervous system disease and genital disease in harbor porpoises (*Phocoena phocoena*) are associated with different herpesviruses. Vet Res. 2016;47:28. doi: 10.1186/s13567-016-0310-8 26861818 PMC4748569

[16] van Beurden SJ, IJsseldijk LL, van de Bildt MWG, et al. A novel cetacean adenovirus in stranded harbour porpoises from the North Sea: detection and molecular characterization. Arch Virol. 2017;162:2035–2040. doi: 10.1007/s00705-017-3310-8 28283815

[17] Endoh D, Mizutani T, Kirisawa R, et al. Species-independent detection of RNA virus by representational difference analysis using non-ribosomal hexanucleotides for reverse transcription. Nucleic Acids Res. 2005;33:e65. doi: 10.1093/nar/gni064 15817564 PMC1074749

[18] Allander T, Emerson SU, Engle RE, et al. A virus discovery method incorporating DNase treatment and its application to the identification of two bovine parvovirus species. Proc Natl Acad Sci. 2001;98:11609–11614. doi: 10.1073/pnas.211424698 11562506 PMC58777

[19] Kruppa J, Jo WK, van der Vries E, et al. Virus detection in high-throughput sequencing data without a reference genome of the host. Infect Genet Evol. 2018;66:180–187. doi: 10.1016/j.meegid.2018.09.026 30292006

[20] Langmead B, Salzberg SL. Fast gapped-read alignment with Bowtie 2. Nat Methods. 2012;9:357–359. doi: 10.1038/nmeth.1923 22388286 PMC3322381

[21] Huson DH, Xie C. A poor man’s BLASTX - high-throughput metagenomic protein database search using PAUDA. Bioinformatics. 2014;30:38–39. doi: 10.1093/bioinformatics/btt254 23658416 PMC3866550

[22] Katoh K, Rozewicki J, Yamada KD. MAFFT online service: multiple sequence alignment, interactive sequence choice and visualization. Brief Bioinform. 2017. 10.1093/bib/bbx108 PMC6781576 28968734

[23] Kumar S, Stecher G, Tamura K. MEGA7: molecular evolutionary genetics analysis version 7.0 for bigger datasets. Mol Biol Evol. 2016;33:1870–1874. doi: 10.1093/molbev/msw054 27004904 PMC8210823

[24] Liebler-Tenorio EM, Ridpath JF, Neill JD. Distribution of viral antigen and tissue lesions in persistent and acute infection with the homologous strain of noncytopathic bovine viral diarrhea virus. J Vet Diagnostic Investig. 2004;16:388–396. doi: 10.1177/104063870401600504 15460320

[25] Cutlip RC, McClurkin AW, Coria MF. Lesions in clinically healthy cattle persistently infected with the virus of bovine viral diarrhea–glomerulonephritis and encephalitis. Am J Vet Res. 1980;41:1938–1941. 6259975

[26] McClurkin AW, Littledike ET, Cutlip RC, et al. Production of cattle immunotolerant to bovine viral diarrhea virus. Can J Comp Med Rev Can Med Comp. 1984;48:156–161. PMC1236029 6326980

[27] Brown C, Baker D, Barker I. The alimentary system. In: Maxie G, editor. Pathology of domestic animals. 5th ed. New York: Elsevier; 2007. p. 2340.

[28] King BF. Comparative studies of structure and function in Mammalian placentas with special reference to maternal-fetal transfer of iron. Am Zool. 1992;32:331–342. doi: 10.1093/icb/32.2.331

[29] Tratschin JD, Moser C, Ruggli N, et al. Classical swine fever virus leader proteinase N^pro^ is not required for viral replication in cell culture. J Virol. 1998;72:7681–7684. 9696875 10.1128/jvi.72.9.7681-7684.1998 PMC110041

[30] Gil LHVG, Ansari IH, Vassilev V, et al. The amino-terminal domain of bovine viral diarrhea virus N^pro^ protein is necessary for alpha/beta interferon antagonism. J Virol. 2006;80:900–911. doi: 10.1128/JVI.80.2.900-911.2006 16378992 PMC1346884

[31] Mayer D, Hofmann MA, Tratschin J-D. Attenuation of classical swine fever virus by deletion of the viral N^pro^ gene. Vaccine. 2004;22:317–328. doi: 10.1016/j.vaccine.2003.08.006 14670312

[32] Lai VCH, Zhong W, Skelton A, et al. Generation and characterization of a hepatitis C virus NS3 protease-dependent bovine viral diarrhea virus. J Virol. 2000;74:6339–6347. doi: 10.1128/JVI.74.14.6339-6347.2000 10864644 PMC112140

[33] Myers TM, Kolupaeva VG, Mendez E, et al. Efficient translation initiation is required for replication of bovine viral diarrhea virus subgenomic replicons. J Virol. 2001;75:4226–4238. doi: 10.1128/JVI.75.9.4226-4238.2001 11287572 PMC114168

[34] Meyers G, Ege A, Fetzer C, et al. Bovine viral diarrhea virus: prevention of persistent fetal infection by a combination of two mutations affecting Erns RNase and N^pro^ protease. J Virol. 2007;81:3327–3338. doi: 10.1128/JVI.02372-06 17215285 PMC1866084

[35] Braun BA, Marcovitz A, Camp JG, et al. Mx1 and Mx2 key antiviral proteins are surprisingly lost in toothed whales. Proc Natl Acad Sci. 2015;112:8036–8040. doi: 10.1073/pnas.1501844112 26080416 PMC4491785

[36] Verhelst J, Hulpiau P, Saelens X. Mx proteins: antiviral gatekeepers that restrain the uninvited. Microbiol Mol Biol Rev. 2013;77:551–566. doi: 10.1128/MMBR.00024-13 24296571 PMC3973384

