# Supplementary material for: An evolutionary divergent pestivirus lacking the Npro gene systemically infects a whale species
Source: Emerg Microbes Infect. 2019 Sep 17;8(1):1383–92. doi: 10.1080/22221751.2019.1664940 (PMC6758615; doi:10.1080/22221751.2019.1664940)
Supplement: Supplemental Material [file TEMI_A_1664940_SM1185.docx]

Figure S1.


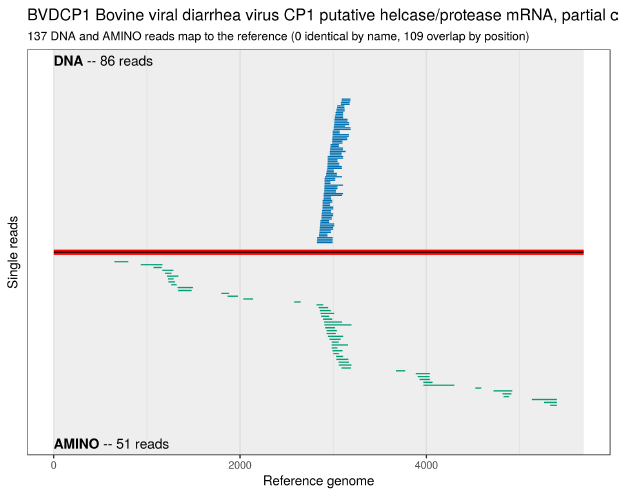


Figure S2.

Table S1.

| **Primer** | **Sequence** |
| --- | --- |
| PhoPeV_1+ | GTATACACAAGGTTAGCTCAATCC |
| PhoPeV _315+ | ACAGTCTGATAGGATGC |
| PhoPeV _544+ | ACCTGATGCTACAATAGTGG |
| PhoPeV _1075+ | TTGTAGGTATGATGAGACGC |
| PhoPeV _1584+ | TTGTAGGCCTAGCAGAGTTT |
| PhoPeV _2072+ | ACTACCCAATGGTATAGAGGC |
| PhoPeV _2525+ | ATAGGAGATGATCTCATGC |
| PhoPeV _2544- | TGCATGAGATCATCTCCTATG |
| PhoPeV _3119+ | TATCAGGTTGAAGCTGCC |
| PhoPeV _3543+ | ATAGAGCAGACAATGAGGC |
| PhoPeV _3720- | AAAGGTTAATATGCACACTATGC |
| PhoPeV _4078+ | ACAAGATGTGGAGCAAGC |
| PhoPeV _4554+ | TTAGGGCACAGACATTGAGC |
| PhoPeV _4571- | TCAATGTCTGTGCCCTAAC |
| PhoPeV _5101+ | TGTTAGTAGTGTCACCC |
| PhoPeV _5612+ | ACTGAGCTACCAAGAAGG |
| PhoPeV _6043+ | AGAAGACCTTACTGAGCAAC |
| PhoPeV _6500+ | TATGACCTATTGCAGGC |
| PhoPeV _6523- | TTTGTGCCTGCAATAGG |
| PhoPeV _7005+ | TTGGCTATGAAGCTCTGTGC |
| PhoPeV _7579+ | GTCAGCACTAGCCAACTA |
| PhoPeV _8081+ | ACAATGGTAGAGCTGGCTG |
| PhoPeV _8634+ | ACCTTAGTGTACTAAGC |
| PhoPeV _8634- | TGAATATCCCACTGGTCC |
| PhoPeV _9142+ | ATCAAGAGGTATGGTTCC |
| PhoPeV _9671+ | GGTACCTACATTGTCAAT |
| PhoPeV _10105+ | TGGTGTTATGGTATCCATAGGC |
| PhoPeV _10562+ | GCAAAGATGAGGCTAGCTAT |
| PhoPeV _10562- | ATAGCTAGCCTCATCTTTGC |
| PhoPeV _11145+ | AACTAGCATATAGGTTCC |
| PhoPeV _11614+ | ATCAAGTCAGGCAGTAGTT |
| PhoPeV _12060- | gggggcctcttggaattgtag |
| PhoPeV_RACE5’_240- | TCCACAGAGCATAACTAGC |
| PhoPeV_RACE5’_315- | GCATCCTATCAGACTGT |

Table S2.

| **GenBank Accession Number** | **Virus** | **Strain** |
| --- | --- | --- |
| MK910227 | PhoPeV | NS170385/Lung |
| MK910228 | PhoPeV | NS170386/Brain |
| KY436034 | LINDA virus | - |
| MH807261 | PPeV | - |
| MH807262 | PPeV | E570 |
| MH807263 | PPeV | H482 |
| JX428945 | AydinPeV | - |
| KM408491 | AydinPeV | Burdur |
| AF144618 | BDV | Reindeer-1_V60-Krefeld |
| KF925348 | BDV | Gifhorn |
| MF102262 | BDV | 35 |
| MG649392 | BDV | 58987 |
| KF918753 | BDV | Aveyron |
| GU270877 | BDV | H2121 |
| AF037405 | BDV | X818 |
| X87939 | CSFV | Alfort187 |
| HQ380231 | CSFV | GZ |
| MK093252 | CSFV | KNU18273 |
| KX576461 | CSFV | 1058 |
| KY849594 | CSFV | 6168 |
| HQ148061 | CSFV | 821 |
| MK405702 | CSFV | Haryana/46 |
| KP233071 | CSFV | HuN23 |
| KC788748 | BVDV-3 | 129 |
| JX469119 | BVDV-3 | JS |
| MH410816 | BVDV-3 | LV03 |
| M31182 | BVDV-1 | NADL |
| KC853441 | BVDV-1 | SuwaCp |
| KR029825 | BVDV-1 | - |
| KX987157 | BVDV-1 | 1170 |
| LC089876 | BVDV-1 | Shitara |
| KT832820 | BVDV-2 | 60767 |
| KT832822 | BVDV-2 | 60779 |
| KC963968 | BVDV-2 | 11F011 |
| AF502399 | BVDV-2 | - |
| U18059 | BVDV-2 | 890 |
| MH806434 | BVDV-2 | 125c |
| GQ888686 | BVDV-2 | JZ05-1 |
| JF714967 | BVDV-2 | - |
| AF144617 | GPeV | H138 |
| KJ660072 | GPeV | PG-2 |
| AY7811525 | PAPeV | Proghorn antelope |
| KJ950914 | RPeV | D23 |
| KY370100 | RPeV | JL |
| MH282908 | BatPeV | GX |
| KX929062 | APPeV | Farm-1 |
| KU041639 | APPeV | BavariaS59 |
| MF167291 | APPeV | NRW-L277 |
| MH715893 | APPeV | GX01 |
| MG792803 | APPeV | JX-JM01 |
| MH509410 | APPeV | KU |
| MH499647 | APPeV | SWU-ZH |
